# Respiratory severity score as a predictive factor for severe bronchopulmonary dysplasia or death in extremely preterm infants

**DOI:** 10.1186/s12887-019-1492-9

**Published:** 2019-04-23

**Authors:** Young Hwa Jung, Jinhee Jang, Han-Suk Kim, Seung Han Shin, Chang Won Choi, Ee-Kyung Kim, Beyong Il Kim

**Affiliations:** 1 Department of Pediatrics, Seoul National University Bundang Hospital, Seoul National University College of Medicine, 82, Gumi-ro 173 beon-gil, Bundang-gu, Seongnam-si, 13620 South Korea; 2 Department of Pediatrics, Seoul National University Children’s Hospital, Seoul, South Korea

**Keywords:** Bronchopulmonary dysplasia, Respiratory severity score, Ventilatory support, Premature infant, Neonatal intensive care

## Abstract

**Background:**

Despite significant advances in neonatology, bronchopulmonary dysplasia (BPD) remains the most common cause of serious morbidity and mortality in premature infants. The aim of the present study was to determine associations between the respiratory severity score (RSS) with death or BPD in premature infants.

**Methods:**

This was a retrospective study conducted between January 2010 and December 2014. We enrolled preterm infants with a gestational age of less than 28 weeks who were supported by mechanical ventilation for more than a week during the first 4 weeks of life. We collected the RSS scores on day of life 2, 7, 14, 21 and 28. The correlations between postnatal RSSs and death or severe BPD were analyzed using multivariate logistic regression.

**Results:**

Of the 138 eligible infants, 66 infants (47.8%) either died or developed severe BPD. The RSS cut-off values for predicting severe BPD or death were 3.0 for postnatal day (PND) 14 with an odds ratio (OR) of 11.265 (*p* = 0.0006, 95% confidence interval (CI), 2.842 to 44.646), 3.6 for PND 21 with an OR of 15.162 (*p* = 0.0003, 95% CI, 3.467 to 66.316), and 3.24 for PND 28 with an OR of 10.713 (*p* = 0.0005, 95% CI, 2.825 to 40.630).

**Conclusion:**

Strong correlations were observed between the RSSs on PND 14, 21, and 28 and death or subsequent severe BPD. The RSS could provide a simple estimate of severe BPD or death., Further research with a larger study population is necessary to validate the usefulness of the RSS for predicting severe BPD or death.

**Electronic supplementary material:**

The online version of this article (10.1186/s12887-019-1492-9) contains supplementary material, which is available to authorized users.

## Background

Bronchopulmonary dysplasia (BPD) is a serious pulmonary condition and one of the most common causes of mortality and morbidity in preterm infants. Although numerous studies have been performed to identify risk factors of BPD to develop preventative therapies, the exact pathogenesis and predictors for BPD remain unclear [1]. In addition, the incidence of BPD has increased over the past 2 to 3 decades despite many advances in neonatal care for preventing BPD, such as gentle ventilation strategies, antenatal steroids, surfactants, and postnatal steroids [2–5]. Because infants with severe BPD require prolonged mechanical ventilation and are associated with the long-lasting burdens of pulmonary and neurodevelopmental sequelae [6–11], research for identifying predictive factors for the earlier diagnosis of BPD severity is valuable; the accurate detection of severe BPD at an early stage may enable the initiation of therapies when they may be more effective and may avoid unnecessary therapies, such as corticosteroids, thereby reducing their potential hazards [12].

The respiratory severity score (RSS) is a simplified severity score consisting of the mean airway pressure (MAP) multiplied by the fraction of inspired oxygen (FiO_2_). The RSS has been used in place of the oxygen index (OI) to assess the need for respiratory support in infants requiring assisted ventilation because the lack of arterial access in infants precludes monitoring the partial pressure of arterial oxygen (PaO_2_) [13]. A previous study revealed a strong association between the RSS and OI at saturation levels of arterial oxygen (SaO_2_) between 88 and 94% in intubated infants [14]. Recently, Malkar et al. demonstrated that an RSS ≥6 on day 30 of life was associated with increased mortality and mechanical ventilation duration in premature infants supported by mechanical ventilation for more than 30 days [15]. The study also suggested the possible usefulness of the RSS as a predictive factor for outcomes in neonatal respiratory conditions.

The objective of the current study was to evaluate the correlation between the RSS and subsequent severe BPD or death.

## Methods

This was a retrospective study conducted in the neonatal intensive care unit at Seoul National University Children’s Hospital between January 2010 and December 2014. Approval for this study was obtained from the Seoul National University Hospital Institutional Review Board.

### Subjects

We enrolled preterm infants with a gestational age of less than 28 weeks who were supported by mechanical ventilation for more than a week during the first 4 weeks of life. The exclusion criteria were as follows: 1) patients with major congenital malformations; 2) patients who died within the first 7 days; or 3) patients who were intubated for ≤7 days or were never intubated.

All infants in this study who needed surfactant instillation were intubated, and minimally invasive surfactant therapy techniques were not applied during the study period. We updated our institutional neonatal resuscitation protocols according to the 2010 American Heart Association guidelines for cardiopulmonary resuscitation and emergency cardiovascular care during the study period [16]. Per the guidelines of neonatal resuscitation, preterm infants who were spontaneously breathing and had respiratory distress were considered for application of positive end expiratory pressure (PEEP) in the delivery room and the selective use of surfactants.

During the first 4 weeks of life, in order to avoid over-distension and atelectasis and consequent injury, we usually start mechanical ventilation rates of 40–60 breaths per minute, inspiration time of 0.25–0.35 s and tidal volume (Vt) of 4–6 ml/kg, and for high frequency oscillation ventilator (HFOV) use of the minimum MAP and amplitude required to achieve clinical goals. In most cases, a PEEP of 4 to 7 cmH_2_O was applied, and FiO_2_ was titrated to achieve an oxygen saturation (SpO_2)_ value between 90 and 95%. The ventilator graphics and lung mechanics were monitored at the bedside, and for some severe cases with weaning difficulty, we adjusted the ventilator settings individually according to the information from the ventilator. A high-frequency oscillation ventilator was usually considered when a plateau pressure of 25 cmH_2_O on pressure-controlled time-cycled ventilation did not reach a sufficient tidal volume or when it was not possible to maintain an SpO_2_ greater than 90% with a FiO_2_ value of at most 0.4–0.6. The ventilator settings were established at the discretion of the clinical teams, and FiO2 was preferentially titrated to minimize oxygen concentration level within a SpO2 range of 90 and 95% before minimizing MAP.

Extubation was considered in patients who showed the following minimal ventilator settings for > 12 h according to the protocol of our center: (1) MAP ≤8 cmH_2_O; (2) FiO_2_ ≤ 0.4; and (3) mandatory respiratory frequency ≤ 35/min.

### Data collection

The charts of the study population were reviewed. Demographic, perinatal and neonatal data were obtained; prenatal factors included preterm premature rupture of the membrane, chorioamnionitis, oligohydramnios, preeclampsia, and antenatal steroid administration. Demographic and neonatal factors included gestational age, birth weight, small for gestational age (SGA), sex, postnatal systemic steroid use, mechanical ventilation duration, and ventilator settings. Ventilator settings on day of life 2, 7, 14, 21, and 28 were collected, and RSSs were calculated. MAP was calculated as MAP = PEEP + ((PIP-PEEP) x (t_i_/t_i_ + t_e_)), where PIP is the peak inspiratory pressure, t_i_ is the inspiratory time, and t_e_ is the expiratory time [17]. Systemic steroid treatment was used as only a rescue therapy for selected severe cases which needed maximal ventilator and oxygen supports according to the American Academy of Pediatrics statement [18]. However systemic steroid treatment could be associated with a reduction in respiratory support, RSSs after systemic steroid administration were excluded from the analysis.

### Outcomes

The primary outcome was death or severe BPD. BPD was defined using the National Institute of Child Health criteria for BPD and graded as mild, moderate, or severe according to the FiO_2_ or positive pressure ventilation (PPV) [19]. Mild BPD was defined as breathing room air, moderate BPD was defined as an FiO_2_ value less than 0.30, and severe BPD was defined as an FiO_2_ value of at least 0.3 or PPV at a postmenstrual age of 36 weeks. Secondary outcomes included the duration of mechanical ventilation or supplemental oxygen, pulmonary arterial hypertension, the duration of hospitalization, and other morbidities such as surfactant use for respiratory distress syndrome, the occurrence of grade 3–4 intraventricular hemorrhage (IVH), cerebral palsy, periventricular leukomalacia, necrotizing enterocolitis (NEC) ≥ stage 2 by Bell’s classification, retinopathy of prematurity (ROP), and culture proven late-onset sepsis.

### Statistical analysis

The chi-square and independent t-tests were used to compare variables. The associations of severe BPD or death with the RSS were analyzed using a multiple logistic regression model. To determine the most predictive time point for severe BPD or death, stepwise backward regression was used. The minimum *p* value approach was applied for selecting the optimal RSS cut-off [20]. Cubic splines were used to allow flexible forms of RSS to be modeled for finding optimal cut-points. A steep and definitive increase in the spline function near a threshold value which is relatively flat before the threshold provides evidence in favor of a cut-point model [21]. Determining the existence of a threshold effect and estimating an optimal cut-point for RSS uses a series of two-sample tests for the multiple possible candidate dichotomizations of RSS. We excluded the outer 10% of RSS distribution to avoid having small numbers in one of the groups following dichotomization, thereby preventing substantial loses in the statistical power. The optimal cut-point was defined as that candidate cut-point with the smallest *p*-value [22]. And cross-validation was performed for measuring the predictive performance of the optimal cut-off value [23]. Statistical analyses were performed using statistical software (SAS, version 9.4; SAS Institute Inc., Cary, NC, USA).

### Ethics statement

The Institutional Review Board of Seoul National University Hospital approved the collection and use of the clinical information of the patients for research purposes before the investigation was started and waived the requirement for informed consent. (IRB No. 1602–026-739).

## Results

### Patients

**Fig. 1:**
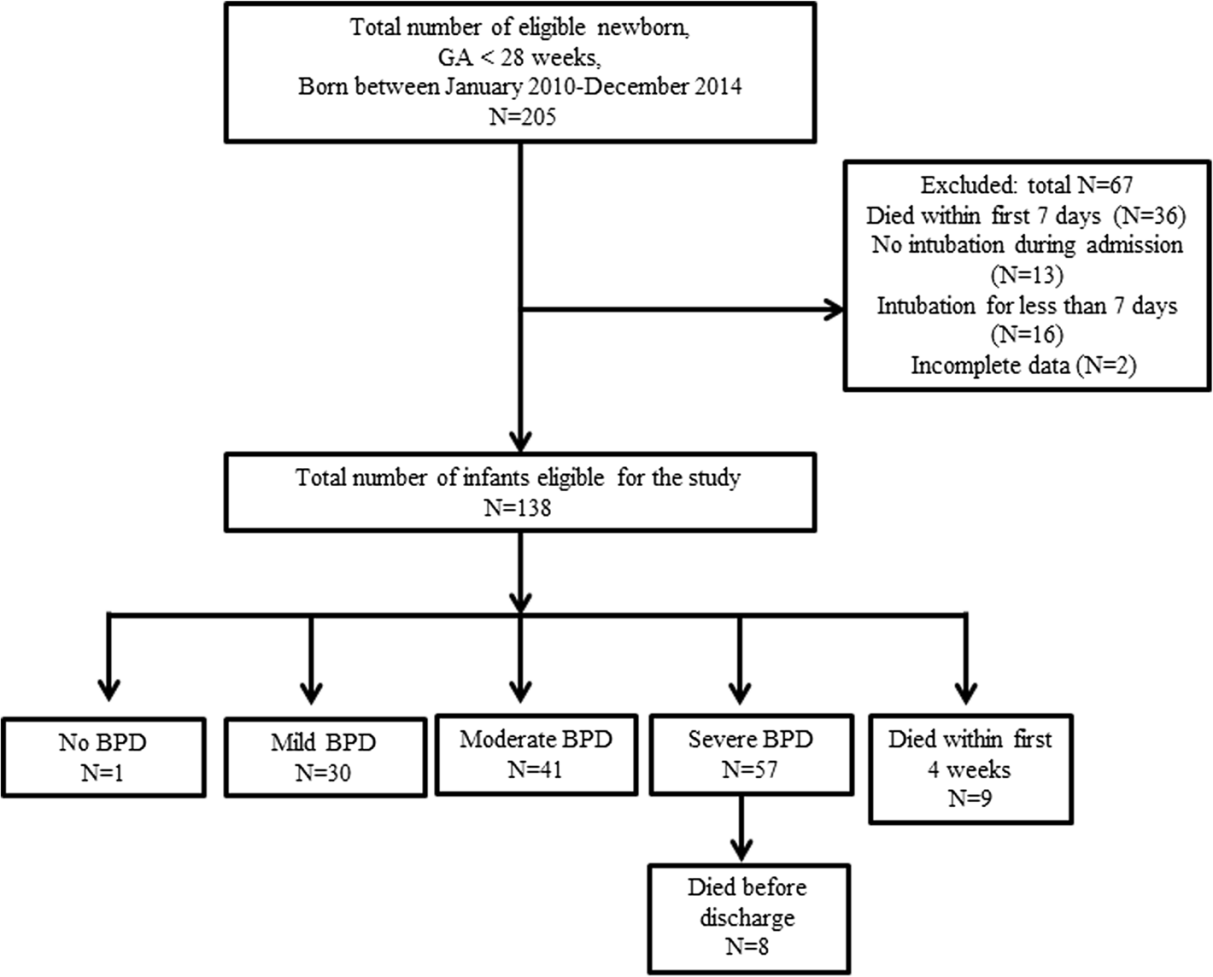
Flow diagram of our study population

A flow diagram of our study population is presented in Fig. 1. During the 5-year study period, a total of 205 neonates born at less than 28 weeks of gestation were eligible for inclusion. After excluding infants who died within the first 7 days, were never intubated, or were intubated for less than a week, a total of 138 patients were enrolled. Of these, 66 (47.8%) infants either died (*n* = 17) or developed severe BPD (*n* = 57). 3 patients required tracheostomy at discharge. Systemic dexamethasone was administered in 7 patients within 4 weeks after birth; all patients required mechanical ventilatory support with FiO_2_ > 0.5 and RSS > 5.0, and a total dose of systemic dexamethasone administered in each patient was 1.1 mg/kg.

### Comparison between the groups with and without severe BPD or mortality

The basal characteristics and clinical data of subjects with and without severe BPD or mortality are compared in Table 1. Infants who died or developed severe BPD had significantly lower gestational ages and birth weights, longer durations of assisted ventilation and supplemental oxygen, increased use of postnatal steroids for BPD, and higher incidences of pulmonary arterial hypertension than survivors without severe BPD. Other morbidities, such as NEC, IVH, ROP, and sepsis were similar between the two groups.

**Table 1:** Demographic and clinical characteristics of the study subjects

	Survivors without severe BPD *N* = 72	Severe BPD or death *N* = 66	*p*-value
Gestational age (week)	26.2 ± 1.2	25.5 ± 1.4	0.002
Birth weight (g)	822.36 ± 226.63	733.79 ± 214.42	0.02
Female/male	35/37	36/30	0.501
SGA	18 (25)	15 (22.7)	0.843
PROM	31 (43.1)	42 (57.5)	0.018
Chorioamnionitis	36 (52.2)	40 (62.5)	0.293
Oligohydramnios	11 (15.3)	15 (22.7)	0.284
Preeclampsia	9 (12.5)	6 (9.1)	0.592
Gestational diabetes	2 (2.8)	6 (9.2)	0.15
RDS	56 (77.8)	60 (90.9)	0.039
NEC stage ≥ II	14 (19.4)	15 (22.7)	0.68
PDA (ligation)	28 (38.9)	32 (48.5)	0.303
Culture proven sepsis	29 (38.9)	34 (51.5)	0.171
Grade III or IV IVH	11 (15.3)	13 (20.0)	0.506
ROP (operation)	34 (47.2)	27 (42.2)	0.606
Postnatal steroids	4 (5.6)	18 (27.3)	0.001
PAH	3 (4.2)	12 (19.7)	0.006
The duration of mechanical ventilation (days)	26.46 ± 16.96	55.98 ± 37.43	< 0.001
The duration of supplemental oxygen (days)	75.26 ± 22.05	100.05 ± 48.74	< 0.001
Length of stay (days)	94.18 ± 18.74	99.71 ± 55.16	0.441

Values are presented as means ± SDs or numbers (%)

*BPD* bronchopulmonary dysplasia, *SGA* small for gestational age, *PROM* premature rupture of membrane, *RDS* respiratory distress syndrome, *NEC* necrotizing enterocolitis, *PDA* patent ductus arteriosus, *IVH* intraventricular haemorrhage, *ROP* retinopathy of prematurity, *PAH* pulmonary arterial hypertension

### Association of RSSs with severe BPD or death

The comparison of RSS values at postnatal days 2, 7, 14, 21 and 28 between the two groups are shown in Table 2. Infants who died or developed severe BPD showed consistently higher RSSs than did those who survived without severe BPD. RSSs at postnatal days 14, 21 and 28 were significantly associated with death or severe BPD. After adjusting for prenatal and postnatal factors that were associated with the development of BPD, the multivariate logistic regression analysis demonstrated that RSSs at postnatal day 14 (odds ratio (OR), 2.135; 95% confidence interval (CI), 1.126 to 4.049), 21 (OR, 2.7; 95% CI, 1.432 to 5.464), and 28 (OR, 3.526; 95% CI, 1.601 to 7.764) remained significantly associated with a higher risk of death or severe BPD.

**Table 2:** The correlation between respiratory severity scores on day of life 2, 7, 14, 21, and 28 and severe bronchopulmonary dysplasia or death

Postnatal day	N	PMA (day) median [min, max]		RSS mean ± SD		Odds ratio	95% Confidence Interval	*P*-value
		Survivors without severe BPD	Severe BPD	Survivors without severe BPD	Severe BPD			
2	135	187 [163, 196]	181 [163, 196]	2.71 ± 2.43	3.45 ± 2.36	1.184	[0.987, 1.42]	0.0684
7	100	192 [169, 201]	186 [168, 201]	2.27 ± 1.09	2.86 ± 1.7	1.424	[0.965, 2.102]	0.0746
14	85	199 [176, 208]	193 [175, 208]	2.41 ± 0.84	4.26 ± 3.31	2.135	[1.126, 4.049]	0.0202
21	84	205 [184, 212]	198 [184, 212]	2.8 ± 0.78	4.28 ± 2.47	2.797	[1.432, 5.464]	0.0026
28	80	213 [190, 222]	207 [189, 222]	2.72 ± 0.64	4.03 ± 2.21	3.526	[1.601, 7.764]	0.0018

*PMA* postmentrual age, *BPD* bronchopulmonary dysplasia, *RSS* respiratory severity score

### RSS cut-off value for predicting death or severe BPD

Higher RSSs at 14, 21 and 28 days of life were significantly associated with a higher risk of death or severe BPD. To develop the RSS as a predictive factor for death or severe BPD, optimal cut-off values were analyzed using a minimum *p* value approach for each of three time points: RSS value was 3.0 for postnatal day 14 with an OR of 11.265 (*p* = 0.0006, 95% CI, 2.842 to 44.646), 3.6 for postnatal day 21 with an OR of 15.162 (*p* = 0.0003, 95% CI, 3.467 to 66.316), and 3.24 for postnatal day 28 with an OR of 10.713 (*p* = 0.0005, 95% CI, 2.825 to 40.630), respectively (Table 3). The sensitivity, specificity, positive and negative predictive values of each RSS values are shown in Table 4. We also performed multivariable logistic regression analyses with including patients who were completely administered the systemic steroid as a secondary sensitivity analysis, the results did not change and the optimal cut-off values for predicting death or severe BPD were same.

**Table 3:** Optimal cut-off of respiratory severity scores for predicting severe bronchopulmonary dysplasia at 14, 21, and 28 days of life

Postnatal day	Cut-point	*p*-value	RSS	Odds Ratio	95% Confidence Interval
14	3	0.0006	RSS < 3.00	0.089	0.022, 0.352
			RSS ≥ 3.00	11.265	2.842, 44.646
21	3.6	0.0003	RSS < 3.60	0.066	0.015, 0.288
			RSS ≥ 3.60	15.162	3.467, 66.316
28	3.24	0.0005	RSS < 3.24	0.093	0.025, 0.354
			RSS ≥ 3.24	10.713	2.825, 40.63

*RSS* Respiratory severity score

**Table 4:** The sensitivity and specificity of respiratory severity scores for predicting severe bronchopulmonary dysplasia or death at 14, 21, and 28 days of life

PND	RSS (95% CI)	AUC	Sensitivity	Specificity	PPV	NPV	*p*-value
14	≥3 (2.842, 44.646)	0.737	57.6	81.63	79.07	61.54	0.0006
21	≥3.6 (3.467, 66.316)	0.724	54.72	86.96	82.86	62.5	0.0003
28	≥3.24 (2.825, 40.63)	0.624	51.85	80.95	77.78	56.67	0.0005

*PND* Postnatal day, *RSS* Respiratory severity score, *AUC* Area under the receiver operating characteristic curve, *PPV* Positive predictive value, *NPV* Negative predictive value

## Discussion

Recently, several trials have focused on the prevention of BPD in very preterm infants, using methods such as steroid administration, inhaled nitric oxide, and stem cell therapy [24–30]. Although those therapies might be beneficial for reducing the incidence of BPD, a lack of evidence exists regarding their safety and effectiveness. Hence, research on preventing BPD must focus on high-risk populations for severe BPD by assessing the balance between potential benefits and harms.

Many clinical models have been developed to predict the risk of BPD. The Eunice Kennedy Shriver National Institute of Child Health and Human Development Neonatal Research Network has developed a predictive model for BPD that is available as a web-based tool to provide estimates of BPD based on demographic variables and respiratory support by postnatal day [31]. This tool might be helpful to identify patients who are at a higher risk for BPD and are most likely to benefit from postnatal preventive treatment. However, Asian race was not considered in this model. Later, Onland et al. reviewed 26 clinical prediction models for BPD in 2013 and concluded that most existing clinical prediction models are poor to moderate predictors of BPD [32]. None of the previous BPD prediction models have been widely adopted.

In the present study, we focused on using the RSS to predict severe BPD or death. RSSs at postnatal days 14, 21, and 28 were significantly associated with death or severe BPD after adjusting for other BPD-related factors. In addition, the RSS could provide an estimate of severe BPD or death at a relatively early stage. At the aspect for prediction, RSS at postnatal day 14 might provide early identification of infants at high risk for BPD or death whilereas RSS at postnatal day 28 might have less meaningful in practice. RSS at postnatal day 21 was the most highly associated with severe BPD or death.

The RSS has been used in several major randomized control trials as a surrogate for the OI in assessing the severity of respiratory illness [25, 33]. The OI is one of well-known respiratory indices for quantifying the severity of oxygenation failure [34, 35]. However, the OI requires an indwelling arterial catheter, which in turn is associated with multiple complications including thromboembolism and ischemic injury. In addition, the OI cannot reflect real-time pulmonary status and needs frequent blood samplings which lead to severe anemia in preterm infants [36]. In current clinical practice, arterial blood sampling is much less common than in the past. As alternative non-invasive methods of assessing the severity of respiratory failure, oxygen saturation index calculated by substituting SpO_2_ for PaO_2_ showed a good correlation with OI [37]. As a strong association between the RSS and the OI in preterm infants under mechanical ventilation at oxygen saturation between 88 and 94% was revealed [14], the RSS can be used instead of the OI when preterm infants maintain at targeted saturation. The RSS can easily determine the need for and assess the response to therapeutic interventions in infants requiring mechanical ventilation.

In this study, we included only patients who required intubated ventilatory support. The intubation rate during the first day of life was 92.6%, which was similar to the value of 89% reported by the Neonatal Research Network, and the moderate to severe BPD rate was 47%, which was similar to or slightly higher than the rates of other studies. We did not use any minimally invasive surfactant therapy techniques during the study period. Although that might have increased the risk of intubation for surfactant instillation, we initially considered the application of PEEP in the delivery room according to the guidelines of the neonatal resuscitation program, as well as the selective use of surfactants. The association between a longer duration of mechanical ventilation and chronic lung disease is well known. Avoiding intubation and using non-invasive ventilator support are necessary to minimize exposure to mechanical ventilation. However, in the Surfactant, Positive Pressure, and Oxygenation Randomized Trial (SUPPORT), 83% of extremely low birth weight infants initially assigned to non-invasive support required endotracheal intubation and mechanical ventilator care [38]. Morley CJ et al. also showed that among preterm infants 25 to 28 weeks in gestational age who had adequate respiratory effort and were assigned to non-invasive support, almost half required endotracheal intubation and mechanical ventilation [39]. In practice, preterm infants with younger gestational ages still require invasive mechanical ventilatory support. The present study showed that the RSS can be used for one of predictors of severity of BPD in these infants who require intubation and mechanical ventilator support. However, there are some limitations that infants who require intubation because of other causes instead of lung conditions, such as airway problems or apnea of prematurity, were included in the study. In addition, RSS can be highly affected on ventilatory support strategies and need to be standardized for validation. Recently, several new modalities for non-invasive respiratory support, such as non-invasive intermittent positive pressure ventilation, and non-invasive neurally adjusted ventilatory assist, have been developed. Those new techniques might provide more sophisticated respiratory support without intubation to extremely preterm infants in near future. Therefore, further investigation would be important to determine the effect of various non-invasive techniques on the outcomes of the present study.

Additionally, because this analysis was retrospective and based on experiences within a single unit, further research with a larger prospective cohort study is necessary to validate the usefulness of the RSS for predicting the outcome at a postmenstrual age of 40 weeks. RSS can be used one of covariates for developing a better clinical model to predict the risk of severe BPD or death.

## Conclusion

In conclusion, the present study demonstrated that there were strong correlations between RSSs at postnatal days 14, 21, and 28 and subsequent severe BPD or death. Furthermore, an RSS ≥ 3.0 at postnatal day 14 and an RSS ≥ 3.6 at postnatal day 21 were reliable values for predicting severe BPD or death. The RSS could provide a simple estimate of severe BPD or death at various postnatal ages and might be helpful for determining the proper time for early interventions.

## Additional file

Additional file 1: Raw data RSS. (XLSX 507 kb)

## Abbreviations

BPD: Bronchopulmonary dysplasia
CI: Confidence interval
FiO2: Fraction of inspired oxygen
IVH: Intraventricular hemorrhage
MAP: Mean airway pressure
NEC: Necrotizing enterocolitis
OI: Oxygen index
OR: Odds ratio
PaO2: Partial pressure of arterial oxygen
PEEP: Positive end expiratory pressure
PND: Postnatal day
PPV: Positive pressure ventilation
ROP: Retinopathy of prematurity
RSS: Respiratory severity score
SaO2: Saturation levels of arterial oxygen
SGA: Small for gestation
