# Correction to: Respiratory severity score as a predictive factor for severe bronchopulmonary dysplasia or death in extremely preterm infants

**DOI:** 10.1186/s12887-019-1642-0

**Published:** 2019-07-31

**Authors:** Young Hwa Jung, Jinhee Jang, Han-Suk Kim, Seung Han Shin, Chang Won Choi, Ee-Kyung Kim, Beyong Il Kim

**Affiliations:** 1 Department of Pediatrics, Seoul National University Bundang Hospital, Seoul National University College of Medicine, 82, Gumi-ro 173 beon-gil, Bundang-gu, Seongnam-si, 13620 South Korea; 2 Department of Pediatrics, Seoul National University Children’s Hospital, Seoul National University College of Medicine, Seoul, South Korea

**Correction to: BMC Pediatr (2019) 19:121**

**https://doi.org/10.1186/s12887-019-1492-9**

Following publication of the original article [1], the authors reported Han-Suk Kim should also be affiliated with Seoul National University College of Medicine. The author group section, above, has been updated.
